# Modelling the cost-effectiveness of cervical cancer screening with HPV self-sampling and molecular triage for women aged 60–69 years

**DOI:** 10.1007/s12672-025-02432-3

**Published:** 2025-05-18

**Authors:** Jasmine Fridljung, Lovisa Bergengren, Linda Ryen, Gabriella Lillsunde Larsson

**Affiliations:** 1 University Health Care Research Centre, Faculty of Medicine and Health, Örebro University, Örebro, Sweden; 2 Department of Women’s Health, Faculty of Medicine and Health, Örebro University, Örebro, Sweden; 3 School of Health Sciences, Faculty of Medicine and Health, Örebro University, Örebro, Sweden; 4 Department of Laboratory Medicine, Faculty of Medicine and Health, Örebro University, Örebro, Sweden

**Keywords:** Cost-effectiveness, Economic evaluation, Cervical screening, HPV, Self-sampling, Methylation, Genotyping, HSIL

## Abstract

**Background:**

Since 2022, self-sampling has been recommended in Sweden’s cervical screening program. Despite still being used primarily for long-term non-attendees, its expected increase in use raises interest in molecular triage methods applicable to self-collected samples. Postmenopausal women face screening challenges due to physiological changes, making this group particularly relevant for evaluating alternative strategies. This study models the cost-effectiveness of different sampling and triage methods in identifying histological high-grade squamous intraepithelial lesions (HSIL) in women aged 60–69 years.

**Methods:**

Using real-world data, this study compares the cost-effectiveness of currently implemented strategy based on professional HPV sampling and combination triage with genotyping and cytology, with modelled strategies based on self-sampling and various molecular triage. The comparison evaluates healthcare resource use and the number of identified histological HSIL cases. The analysis focuses on a single screening cycle, re-testing of invalid samples or screening-positive/triage-negative women are not addressed.

**Results:**

Screening with molecular triage either leads to decreases in effect, i.e. fewer histological HSIL identified, or significant cost increases due to higher rate of HPV-positive screening samples and higher number of colposcopy follow-ups.

**Conclusions:**

Molecular triage, whether used with self-sampling or professional sampling, does not appear cost-effective for identifying HSIL in this age group compared to the current screening strategy.

## Background

Since 2022, the HPV screening recommendations from the Swedish National Board of Health and Welfare are that self-sampling can be offered as an option to all screening participants in the cervical screening program [1, 2]. In Sweden, self-sampling is still mainly used as a way of reaching screening non-attendees. However, with expected future increase in the use of self-sampling, there is an interest in triage methods that can be performed directly on HPV-positive self-collected samples. Since the national transition from cytology-based screening to HPV-based screening, cytology is still used together with HPV genotyping as a combination triage method to increase screening specificity [3, 4]. However, cytological triage cannot be performed directly on self-collected samples and requires HPV-positive women to undergo a second professional screening sampling, which in Sweden, is mainly performed by midwives.

Evaluating self-sampling and molecular triage in postmenopausal women is of particular interest, as this group differs from younger cohorts in several ways. Hormonal changes associated with post menopause leads to atrophic vaginal and cervical epithelia, as well as a retracted transformation zone, making cervical sampling more challenging. Vaginal self-sampling, however, is sufficient to produce a sample for HPV testing and molecular triage and can be performed by the woman in her own home. Additionally, cytology-based testing has shown unsatisfactory results in detecting cervical dysplasia in this age group. Screening among older women remains crucial, as approximately 30% of cervical cancer cases occur in women over 60 years of age, with high mortality rates [5–9].

Previous cost-effectiveness analyses (CEA) show that self-sampling may be more cost-effective than professional sampling when analyzing additional cost per additional quality-adjusted life year [10] and detection of cervical intraepithelial neoplasia grade 2 or higher [11]. Previous studies also show that self-sampling has equivalent accuracy [12–14] and can lead to higher participation levels [15, 16]. A recent study, on the same screening cohort analyzed in this CEA, shows that women in this age group are positive towards and have confidence in self-sampling, which is important since this screening test is the last before they exit from the screening program [17].

Methylation and genotype-based triage, hereafter referred to as molecular triage, are described as potential alternatives to cytology [18–21] and can be used directly on self-collected samples. HPV genotypes are classified according to risk for development of cell dysplasia and cancer [21–23]. There are 13 genotypes defined as high-risk or potentially high-risk according to WHO/IARC, but many test panels include 14 HPV genotypes, with differences in oncogenic risk within the group [24]. HPV16, 18, and 45 have the highest risk scores followed by HPV 31, 33, 52, and 58. Besides HPV genotyping, methylation of human genes FAM-19A4 and hsa-miR124–2 has been suggested for increased specificity in HPV-based screening [20, 25]. Molecular triage methods can be implemented as single triage methods or in various combinations.

Cost-effectiveness is a relative concept that considers both the costs and effects of interventions, providing knowledge about the expected impact of choosing one intervention, in this setting one sampling and triage strategy, over another [26]. Insufficient screening specificity leading to unnecessary colposcopy follow-ups is not only costly and resource demanding for the healthcare sector, but it can also entail societal costs by being time-consuming and anxiety-provoking for the screening participants [27–29].

Based on data from a Swedish population-based cervical screening study (the Self-Sampling Study), the aim of this analysis is to compare the cost-effectiveness of the currently used sampling and triage methods in the Swedish national cervical screening program for women aged 60–69 years, with modelled self-sampling and molecular triage strategies, regarding their resource use and ability of identifying histological high-grade squamous intraepithelial lesions (HSIL) [30]. This age group is particularly relevant in the Swedish context due to recent recommendations for these women to undergo catch-up HPV screening, due to not previously being HPV-tested [2].

This study provides valuable insights for optimizing future screening strategies. By evaluating the cost-effectiveness of these strategies, the study can inform policy decisions on resource allocation and program design.

## Methods

### Study population

The study population consists of all women in Region Örebro County eligible for screening between the years 2018 and 2020 and who were between 60 and 69 years old at that time. This resulted in a total of 2,258 women included in a Swedish population-based cervical screening study on self-sampling and molecular triage. For more details on the study population, we refer to the primary study (see Ref. [30]).

Our aim was to study women in this age group, as they were not previously HPV-tested (although having participated in routine screening with primary cytology) and because HPV test now is recommended as an exit test in Sweden [2, 31]. The participants in the study attended the regular screening program with professional sampling and, additionally, performed vaginal self-sampling. Hence, all participants were invited to contribute with both sample types and thereby enable comparisons between professional sampling with HPV and cytology triage and self-sampling with HPV and different molecular triages.

The vaginal Aptima® Multitest Swab Specimen Collection Kit was used for self-sampling (Aptima Hologic, Marlborough, MA, USA). Paired sample types (i.e. collected both professionally and by self-sampling) were then analyzed for HPV using the screening test HPV Aptima assay (Aptima, Hologic) detecting mRNA from 14 genotypes (HPV 16, 18, 31, 33, 35, 39, 45, 51, 52, 56, 58, 59, 66, and 68). This is a validated screening HPV test used in routine clinical diagnostics in the Region of Örebro county as well as in other settings. Samples positive for mRNA were genotyped with the Anyplex II HPV28 assay detecting HPV DNA from 28 HPV genotypes (Seegene, Seoul, Korea), and they were analyzed for methylation of human genes FAM19 A4 and miR124–2 using the QIAsure test (Qiagen, Hilden, Germany). Data include all HPV-screened women from both professional sampling and self-sampling generated in the Self-Sampling Study.

### Screening strategies

Six strategies (Table 1) were included in this CEA (strategies A-D2); the currently implemented strategy in Sweden, i.e. professional sampling, HPV screening test, genotyping, and cytology triage [2], and the modelled strategies that all begin with self-sampling screening followed by various molecular triage methods. This study analyzes a single screening cycle; re-testing of invalid samples or screening-positive/triage-negative women are not addressed. All strategies are based on data from analyses performed on HPV-samples from the same women (professionally collected and self-collected) [30].

**Table 1:** Included HPV screening strategies

Current strategy	Modelled strategies				
Professional HPV sampling	Self-collected HPV sampling				
HPV positive	HPV positive				
A. Full genotyping and cytology	B. Methylation	C1. Extended genotyping	C2. Full genotyping	D1. Extended genotyping and methylation	D2. Full genotyping and methylation
HPV positive and ASCUS +	Methylation positive	HPV positive	HPV positive	HPV positive and methylation positive	HPV positive and methylation positive
Follow up with colposcopy					
HSIL					

All included screening strategies. A is the currently implemented strategy, while strategies B-D2 are hypothetical, modelled strategies using self-sampling and various triage methods. Full genotyping includes 14 genotypes; 16, 18, 31, 33, 35, 39, 45, 51, 52, 56, 58, 59, 66, and 68. Extended genotyping includes 7 genotypes; 16, 18, 31, 33, 45, 52, 58

**A.** *Current Swedish strategy.* Current strategy includes professional sampling by a midwife. The HPV screening test detects viral mRNA (HPV Aptima, Hologic). All HPV-positive samples are genotyped with a DNA-based HPV test (Allplex HPV HR detection, Seegene). Genotype positive for any of the 14 genotypes (referred to as full genotyping) are further triaged with cytology. Women with any form of aberrant cytology, Atypical Squamous Cells of Undetermined Significance (ASCUS) or more severe (ASCUS +) are followed up with colposcopy and potential cervical biopsy. Histology diagnosis will determine possible treatment for dysplasia according to national guidelines.

**B.** *Self-sampling, HPV screening test followed by methylation analysis as triage.* This strategy included the same HPV screening test as in strategy A, but performed on self-collected samples, and followed by a DNA-based methylation assay (QIAsure, Qiagen). Women with a methylation-positive test (one or both genes) were modelled as subject to colposcopy follow-up according to the same criteria for colposcopy referral as in strategy A.

**C1/C2.** *Self-sampling, HPV screening test followed by HPV genotyping as triage.* This strategy included the same HPV screening test as in strategy A but performed on self-collected samples and followed by DNA-based genotyping test (Allplex HPV HR detection, Seegene). Women with a positive test for either HPV genotypes 16, 18, 31, 33, 45 52, 58 (C1, extended genotyping) or any of the 14 genotypes defined as high risk (C2, full genotyping) were modelled as subject to colposcopy follow-up according to the same criteria for colposcopy referral as in strategy A.

**D1/D2.** *Self-sampling, HPV screening test followed by a combination triage considering results from both HPV genotyping and methylation.* This strategy included the same HPV screening test as in strategy A, performed on self-collected samples and followed by DNA-based genotyping (Allplex HPV HR detection, Seegene) and a DNA-based methylation assay detecting genes FAM19 A4/miR124-2 (QIAsure, Qiagen). Women with a positive test for HPV genotypes 16, 18, 31, 33, 45 52, 58 in combination with a methylation-positive test (one or both genes) (D1, extended genotyping), or women positive for any of the 14 genotypes defined as high risk in combination with a methylation-positive test (one or both genes) (D2, full genotyping), were modelled as subject to colposcopy follow-up according to the same criteria for colposcopy referral as in strategy A.

### Analytical framework

This CEA was conducted from a healthcare perspective using a decision tree for one screening cycle. We include costs and effects over a single screening cycle to assess which screening and triage strategy most effectively identifies cases of HSIL. This represents a snapshot of the screening process, with long-term effects not included. In the decision tree, the patient flows were designed based on transition probabilities derived from the Self-Sampling Study [30]. When comparing two strategies with respect to costs and effects, four outcomes are possible, namely:

1. Higher costs and lower effects, i.e. strongly dominated and excluded from further analysis.
2. Higher costs and higher effects, subject to further analysis.
3. Lower costs and higher effects, subject to further analysis.
4. Lower costs and lower effects i.e. weakly dominated. This strategy can be excluded or, if aiming for cost saving, be subject to further analysis [32].

In cases where two strategies produce identical outcomes, they cannot be categorically assigned to a specific quadrant. Further, if strategies have the exact same effectiveness they cannot be compared in an incremental analysis, and in that case, the least costly one should be recommended. With more than two strategies that are mutually exclusive, all strategies are traditionally compared in pairs. All strategies will be ordered in ascending order of effect and dominated alternatives will be removed before any further calculations [33].

**Fig. 1:**
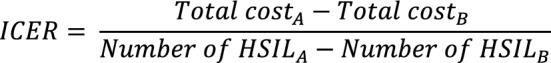
Incremental cost-effectiveness ratio (ICER*).* An ICER is calculated to compare costs and effects between different strategies

The comparison between strategies is then performed with respect to costs and effects by calculating the incremental cost-effectiveness ratio (ICER). An ICER is calculated by dividing the difference in costs between two interventions by the difference in effects. The ICER will thus represent the cost per additional unit of effect that is achieved if choosing one strategy over another. In this CEA, costs were based on total costs for one screening cycle, and effectiveness was represented by the total number of histological HSIL detected. Since the number of participants was the same in all strategies, the results are not affected by whether total cost or average cost is used. The ICER in this setting is defined as seen in Fig. 1 above:

### Data

Cost parameters were estimated by the department of Laboratory medicine at Örebro University Hospital based on actual resource use for triage analysis and self-sampling kits (as opposed to charged prices). Data on wages for midwives were collected from Region Örebro County’s administrative sources, and colposcopy costs were collected from the Cost per Patient database (Table 2). Transition probabilities presented in the table represent the likelihood of moving from one health state to another in a specified period. Transition probabilities used in the model were derived from the earlier described study on self-sampling and molecular triage methods (see Appendix A for complete model) [30].

**Table 2:** Input data into analytical model

Costs			
Examination	Unit costs (SEK)	Cost components (SEK)	Source
Professional sampling	334	Screening visit including wage and material costs (114) *, HPV Aptima analysis, wages, and departmental overhead costs (OH) (220) **	*Administrative source **Department of laboratory medicine
Self-sampling	275	Material (10), postal distribution (45), HPV Aptima analysis, wages, and OH (220)	Department of laboratory medicine
Cytology	432	Laboratory analysis including laboratory staff costs and OH	Department of laboratory medicine
Methylation	1637	Laboratory analysis including laboratory staff costs and OH	Department of laboratory medicine
Genotyping (ext./full)	402	Laboratory analysis including laboratory staff costs and OH	Department of laboratory medicine
Colposcopy	6989	Includes only the colposcopic examination (not histological sampling)	Cost per Patient database
Transition probabilities			
Procedure			Positivity rates
Professional sampling (PS)			0.03 (77/2258)
Self-sampling (SS)			0.13 (284/2258)
Full genotyping after positive PS			0.78 (60/77)
Cytology triage after positive PS and full genotyping			0.62 (37/60)
Methylation triage after positive SS			0.60 (169/284)
Extended genotyping triage after positive SS			0.48 (136/284)
Full genotyping triage after positive SS			0.80 (228/284)
Combination triage (extended genotyping) after positive SS			0.29 (83/284)
Combination triage (full genotyping) after positive SS			0.48 (137/284)
Procedure			Rate of invalids
PS screening			0 (1/2258)
SS screening			0.05 (110/2258)
Methylation analysis after positive PS			0.61 (47/77)
Methylation analysis after positive SS			0.01 (4/284)

Transition probabilities derived from the Self-Sampling Study. Combination triage: positive extended genotyping (16, 18, 31, 33, 45 52, 58) or full genotyping (16, 18, 31, 33, 35, 39, 45, 51, 52, 56, 58, 59, 66, and 68) followed by positive methylation. Numbers presented as shares and absolute numbers. All costs in SEK, 2022 price level

## Results

### Base case analysis

All modelled strategies were associated with increased total costs compared to the current strategy, and two of the modelled strategies also imposed a decrease in effect, i.e. fewer identified cases of histological HSIL. The number of identified histological HSIL decreased by 29% or remained unchanged. Total costs increased between 30 and 118% compared to the current strategy, mainly due to the modelled number of colposcopy follow-ups that increased between 124 and 516%. For results like this, where all strategies imposed increased costs without contributing an increase in effect, incremental analysis will not be applicable, and none of the modelled strategies would be recommended over the currently implemented strategy (Table 3).

**Table 3:** All screening strategies in base-case analysis ordered in ascending order of effect

Screening strategy	Total cost (SEK)	N HSIL	N colposcopies	Incremental analysis
A. Current strategy	1 069 639	7	37	
D2. Full genotyping and methylation	1 894 368	7	137	Weakly dominated
B. Methylation	2 266 999	7	169	Weakly dominated
C2. Full genotyping	2 328 610	7	228	Weakly dominated
D1. Extended genotyping and methylation	1 391 580	5	83	Strongly dominated
C1. Extended genotyping	1 685 622	5	136	Strongly dominated

All costs in SEK, 2022 price level

### Repeating the analysis solely considering positives in professional sampling

The modelled self-sampling strategies had a larger share of HPV-positive screening participants proceeding to triage, 13% positives in self-sampling (modelled strategies) compared to 3% positives in professional sampling (current strategy). We repeated the analysis without self-sampling to evaluate if molecular triage after positive professional sampling is a cost-effective option, considering its potential to reduce analysis subjectivity and streamline the workflow. A repetition of the analysis was therefore conducted considering only positives in professional sampling. The cost of self-sampling was replaced with the cost of professional sampling.

Results of this analysis are presented in a table of costs and effects (Table 4). It is worth noting that four out of five modelled strategies failed to detect as many histological HSIL as the current screening program (details about analysis results for the identified histological HSIL cases can be found in Appendix B). However, these four strategies were associated with decreases in total costs. From a purely health economics perspective, strategies with lower expected effectiveness and lower expected costs could be of interest for further analysis and presentation to policymakers if the aim is to reduce costs while accepting lower effectiveness. However, in this setting, where a screening strategy is already implemented, saving costs by accepting lower effectiveness is not considered an option for the decision-makers. Hence, strategies with expected lower effectiveness were not further analyzed. Discussions about potentially extending screening intervals and similar adjustments apply to younger and vaccinated cohorts who have only recently entered the screening program.

**Table 4:** All screening strategies in repeated analysis ordered in ascending order of effect

Screening strategy	Total cost (SEK)	N HSIL	N colposcopies	Incremental analysis
A. Current strategy	1 069 639	7	37	
C2. Full genotyping	1 204 466	7	60	Weakly dominated
D2. Full genotyping and methylation	1 002 159	5	17	Weakly dominated
B. Methylation	1 020 001	5	20	Weakly dominated
C1. Extended genotyping	1 029 741	5	35	Weakly dominated
D1. Extended genotyping and methylation	912 311	4	10	Weakly dominated

All costs in SEK, 2022 price level

### Sensitivity analysis

In a sensitivity analysis, input parameters can be varied to gain knowledge about the robustness of the results and about what degree of parameter variations would be required to change the recommendation. First and foremost, the parameter uncertainty should be assessed, and parameters relevant for variation should be identified. In this analysis, uncertainties around medical parameters were relatively small as they are the result of primary laboratory analyses performed on samples from the same cohort. Regarding cost parameters, the uncertainties for most of the parameters were also relatively small as they represented resource use rather than charged prices, which is desirable in health economic evaluations. The cost items assessed as still important to vary were the costs for genotyping and methylation. These cost calculations were not derived from a screening setting, and large-scale use would presumably lead to lower costs. For methylation testing, large-scale implementation could potentially reduce costs due to different pricing structures and procurement processes. For genotyping, large-scale use could potentially lead to the selection of an alternative genotyping method that allows for broader application. It was therefore deemed relevant to analyse whether a reduction in these triage costs would impact the recommendation.

Strategies that did not impose any decreases in effect and had the smallest cost increases compared to the current strategy in the base case and repeated analysis, namely strategies D2 (base case) and C2 (repeated), are affected by the costs for genotyping and methylation. Therefore, these two strategies were chosen for sensitivity analysis. Results showed that even when triage costs were varied down to zero, a scenario where triage tests would be free of charge for the healthcare providers, it would not change the recommendation. Both in the base case and in the repeated analysis, the modelled strategies were still characterized by increased costs compared to the currently implemented strategy (Table 5). Hence, the effect of a price reduction did not outweigh the increase in the number of modelled colposcopies, as the rise in colposcopies is the primary driver of the cost increase.

**Table 5:** Sensitivity analysis, excluding triage costs for specific strategies in base case and in repeated analysis

Strategy	Total cost (triage cost included)	Total cost (triage cost excluded)	Strategy	Total cost (triage cost included)
Base case: self-sampling and molecular triage				
D2: full genotyping, methylation	1 894 368	1 578 443	A: Current strategy	1 069 639
Repeated analysis: professional sampling and molecular triage				
C2: full genotyping	1 204 466	1 173 512	A: Current strategy	1 069 639

## Discussion

This CEA was based on effect data from a real-world single-group study. The included costs are mainly based on estimations of actual resource use rather than prices, which is desirable in health economic evaluations. Base case analysis indicated that self-sampling and molecular triage, in various combinations, generally increased costs for screening women 60–69 years included in this analysis. For two modelled strategies, where extended genotyping was used as the sole triage method or in combination with methylation, the effect was also negatively impacted. Repeated analysis, considering only professional sampling, lowered costs in most strategies, mainly due to a significantly lower rate of colposcopies. However, the reduced costs came at the price of fewer cases of identified histological HSIL The only strategy in the repeated analysis that did not lead to inferior effect was full genotyping as triage. That strategy was 13% more expensive, however, and came with a 62% increase in the number of colposcopies compared to the current strategy. Given increased costs without increased effectiveness, there was no reason to believe that any molecular triage strategy was cost-effective for this age group. In a sensitivity analysis, costs for triage tests were varied to assess whether the recommendation would change with lower costs for genotyping and methylation. Results indicated that triage costs were of less importance for the recommendation, and that the main cost driver was the increased number of colposcopy follow-ups.

Since the transition to HPV-based screening, the problem of increased colposcopy follow-ups has become evident. This study, when utilizing HPV screening in the form of self-sampling followed by molecular triage (base case scenario), also illustrated this consequence. When HPV screening was changed to professional sampling (repeated analysis), this increase was not as evident, albeit with great uncertainty related to the high proportion of invalid methylations. Colposcopy follow-ups not only burden the healthcare system but also the individuals, in the form of health losses. The procedure itself can be challenging to perform on postmenopausal women, often yielding inconclusive results that require further biopsies, further escalating costs [34]. Another potential harm seen is the anxiety women can experience having to go through this procedure [35]. Altogether, strategies that potentially increase colposcopy rates might underestimate the true societal cost if individual health losses are not included.

Regarding the time perspective of our analysis, we have chosen to focus on a single screening cycle, including only the costs and effects within this timeframe. This approach is motivated by our objective: to evaluate different screening and triage methods, and their ability to identify HSIL, from a health economic perspective. If we were to extend the analysis to include long-term costs and effects, we would inevitably introduce additional uncertainties that are not directly related to the screening and triage methods themselves.

A limitation in this study is the invalid methylation results from professional sampling that impacted the output in the repeated analysis. A substantial number of samples in the baseline data were invalid due to lack of DNA. Interestingly, this was not a problem in self-collected samples (base case). A possible explanation might be that professionally collected samples were sparse in cell material and needed to be sufficient for cytology analysis and DNA HPV genotype analysis. However, since HPV genotyping could be performed, invalids would probably not be a major problem in professional sampling in a clinical setting where samples hold sufficient DNA. The threshold in the used methylation kit for positive methylation results could also have influenced the number of invalids. Another potential limitation is that there is no established threshold for what cost per additional HSIL identified that is deemed cost-effective. The findings here are however not affected by that limitation, as the most effective strategy also is the one with the lowest cost. However, if the aim primarily would have been cost savings a threshold would have been necessary to evaluate whether a potential loss in effect when choosing one strategy over another justifies savings in costs.

The baseline data underlying this study showed that vaginal self-sampling is as effective as professional sampling for detecting histological HSIL in this group of women, using HPV screening. However, despite the fact that this age group is positive towards self-sampling [17], in a real screening setting it is not certain that all women accepting professional sampling would accept self-sampling, and the groups actually screened could differ if self-sampling was implemented instead of professional sampling. The number of HPV-positives was considerably higher among self-collected samples compared to professionally collected samples. The same discrepancy of HPV positivity between sampling methods (using Aptima HPV assay as the screening test) has been previously reported and speculated to be a result of methodological differences or additions of false positives due to aerosol transfer during an initial heating step [14, 36]. Other studies based on different HPV assays and where samples were professionally collected report HPV positivity for this older age group of women similar to what we found among our professionally collected samples [31, 37]. Despite the high HPV-positive rate in self-collected samples the amount of histological HSIL’s were equal in both sample types.

Also, a high number of invalids were present among the self-collected samples. Auvinen et al. report a similar finding for this age group and provide the explanation that mucus in the samples might be the cause for invalid results [36]. Therefore, based on both rates of positives and invalids, the use of Aptima HPV assay in a self-sampling setting among older women needs to be thoroughly evaluated regarding both effects and costs.

## Conclusion

In this analysis, screening with molecular triage either leads to a decrease in effectiveness, i.e. fewer identified cases of histological HSIL, or significant cost increases due to the high rate of HPV-positives and the high number of colposcopies. Consequently, molecular triage, whether screening is based on self-sampling or professional sampling, does not appear to be cost-effective for the age group 60–69 years, compared to the currently implemented strategy. Factors related to molecular testing in a screening triage setting, such as objectivity in testing methods and streamlining in laboratory settings, could be a motive to further analyze other laboratory methods for molecular triage.

## Data Availability

Data is provided within the manuscript.

## Appendix

### B. Outcomes for known HSIL cases detected in the current strategy

**Table:**

Outcomes for known HSIL cases detected in current strategy			
Self-sampling screening		Professional sampling screening	
Molecular triage outcomes	Not detected in	Molecular triage outcome	Not detected in
1) HPV 16, 35, methylation positive		1) HPV 16, 35, methylation positive	
2) HPV 16, 35, 39, methylation positive		2) HPV 16, 35, 39, methylation positive	
3) HPV 35, methylation positive	Strategies C1, D1	3) HPV 35, methylation positive	Strategies C1, D1
4) HPV 16, methylation positive		4) HPV 16, methylation positive	
5) HPV 68, methylation positive	Strategies C1, D1	5) HPV 68, methylation analysis invalid	Strategies B, C1, D1, D2
6) HPV 52, methylation positive		6) HPV 52, methylation positive	
7) HPV 31, methylation positive		7) HPV 31, methylation analysis invalid	Strategies B, D1, D2
